# Mitochondrial mRNA transcripts predict overall survival, tumor recurrence and progression in serous ovarian cancer: Companion diagnostics for cancer therapy

**DOI:** 10.18632/oncotarget.19963

**Published:** 2017-08-06

**Authors:** Federica Sotgia, Michael P. Lisanti

**Affiliations:** ^1^ Translational Medicine, School of Environment & Life Sciences, Biomedical Research Centre, University of Salford, Greater Manchester, United Kingdom

**Keywords:** ovarian cancer, mitochondrial biomarkers, treatment failure, relapse, recurrence

## Abstract

Here, we performed a systematic analysis to discover new biomarkers of overall survival and tumor progression in ovarian cancer patients. More specifically, we determined whether nuclear-encoded mitochondrial genes related to mitochondrial biogenesis and function are effective in predicting clinical outcome in ovarian cancer. As a consequence, we are able to provide *in silico* validation of the prognostic value of these mitochondrial markers, in a well-defined population of ovarian cancer patients. Towards this end, we used a group of *N*=111 ovarian cancer patients (serous type; stage III), with optimal de-bulking. Importantly, in this group of cancer patients, CA125 and PCNA (conventional markers) were associated with poor overall survival, as would be expected. Using this approach, we identified >100 new individual mitochondrial gene probes that effectively predicted significantly reduced overall survival, with hazard-ratios (HR) of up to 3.68 (*p* < 9.8e-05). These mitochondrial mRNA transcripts included membrane proteins, chaperones, anti-oxidant enzymes, as well as mitochondrial ribosomal proteins (MRPs) and key members of the OXPHOS (I-V) complexes. Based on this bioinformatics analysis and in silico validation, we conclude that mitochondrial biogenesis and OXPHOS should both be considered as new therapeutic targets, for the more effective treatment of human ovarian cancers. The mitochondrial biomarkers that we have identified could also be employed as new companion diagnostics to assist oncologists in: i) more accurately predicting clinical outcomes and ii) improving the response to therapy, in ovarian cancer patients.

## INTRODUCTION

Drug-resistance dramatically limits the effectiveness of most cancer therapies, and especially for ovarian cancer patients [1, 2]. As such, treatment failure remains a significant barrier to successful cancer therapy and precision medicine [3, 4]. As a result, new biomarkers are urgently required for the treatment stratification of ovarian cancer patients, into different risk sub-groups at diagnosis (high-risk *versus* low-risk) [5].

In this report, we tested the hypothesis that mitochondrial markers might have prognostic value for the identification of high-risk ovarian cancer patients, with increased progression and poor overall survival. For this purpose, we used a data-mining and informatics strategy to determine the potential effectiveness of mitochondrial gene transcripts, in predicting clinical outcome.

Our results indicate that >100 mitochondrial gene probes can be used individually or in various combinations, to predict poor overall survival in ovarian cancer patients. Based on these current findings, we speculate that mitochondrial biogenesis and/or OXPHOS could be targeted therapeutically to prevent ovarian cancer recurrence and extend overall survival.

## RESULTS

### Prognostic value of conventional markers (CA125 and PCNA) in the patient population

**Figure 1:**
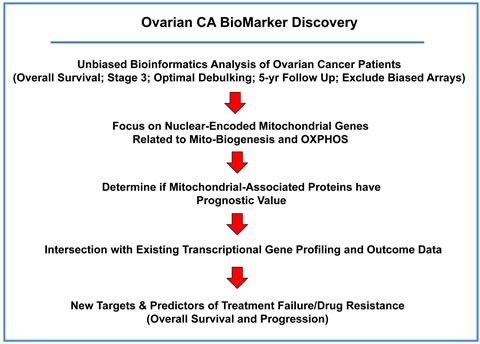
Summary illustrating our systematic approach to ovarian cancer biomarker discovery For this analysis, we chose to focus on serous ovarian cancer patients, with optimal de-bulking, and 5-years of follow-up data (*N* = 111). In this context, we evaluated the prognostic value of mitochondrial markers for predicting overall survival (OS), progression-free survival (PFS) and post-progression survival (PPS).

To identify novel biomarkers for ovarian cancers, we employed publically available transcriptional profiling data from the tumors of patients with serous ovarian cancer (stage III), with optimal de-bulking, low CA125 levels at diagnosis, and 5-years of follow-up data (Figure 1).

**Figure 2:**
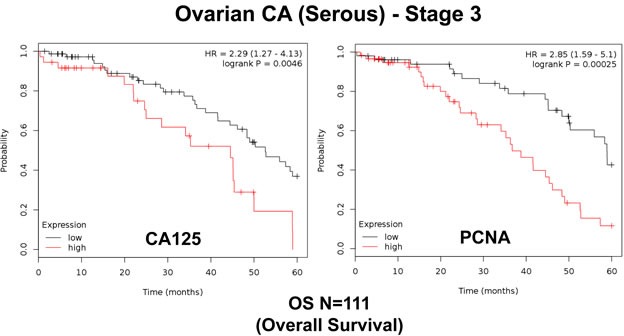
Traditional markers (CA125 and PCNA) predict poor overall survival in ovarian cancer patients We assessed the predictive value of CA125 and PCNA in *N* = 111 ovarian cancer patients, with optimal de-bulking. Note that high transcript levels of CA125 and PCNA are associated with significantly reduced overall survival.

First, we assessed the prognostic value of CA125 in this context. The results of this analysis are shown in Figure 2 and Table 1. Note that the hazard-ratio (HR) for CA125 was 2.29 (*p* = 0.005) for overall survival (OS). As proliferative markers are often used as key endpoints in Phase II clinical trials, we next assessed the prognostic value of Ki67 and PCNA. Figure 2 and Table 2 both show the prognostic value of these markers. The results with Ki67 were not significant, but PCNA showed a hazard-ratio of 2.85 (*p* = 0.00025). Similarly, we determined the utility of macrophage-specific markers of inflammation. However, Table 3 shows that that CD68 and CD163 did not show significant prognostic value.

**Table 1:** Prognostic value of CA125 in ovarian cancer

Gene Probe ID	Symbol	Hazard-Ratio	Log-Rank Test
201383_s_at	CA125	2.29	0.005
220196_at	CA125	1.35	0.29
201384_s_at	CA125	0.66	0.15

**Table 2:** Prognostic value of KI67 in ovarian cancer

Gene Probe ID	Symbol	Hazard-Ratio	Log-Rank Test
212020_s_at	MKI67	1.50	0.17
212023_s_at	MKI67	1.47	0.21
212021_s_at	MKI67	0.75	0.44
212022_s_at	MKI67	0.65	0.16

**Table 3:** Prognostic value of PCNA and markers of inflammation in ovarian cancer

Gene Probe ID	Symbol	Hazard-Ratio	Log-Rank Test
201202_at	PCNA	2.85	0.0003
217400_at	PCNA	2.48	0.001
215049_x_at	CD163	1.43	0.20
203645_s_at	CD163	1.45	0.19
216233_at	CD163	0.47	0.024
203507_at	CD68	0.57	0.095

Thus, a subset of conventional markers (CA125 and PCNA) can be used to predict overall survival in ovarian cancer patients.

### Prognostic value of individual mitochondrial markers

Our hypothesis is that increased mitochondrial biogenesis drives poor overall survival in ovarian cancer patients. To directly test this hypothesis, we next determined the prognostic value of a series of mitochondrial markers.

**Figure 3:**
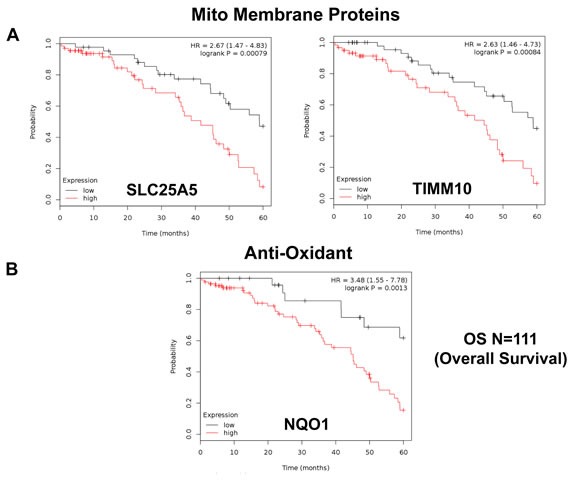
Mitochondrial membrane proteins and NQO1 are associated with poor clinical outcome in ovarian cancer patients **A.** Note that that high transcript levels of SLC25A5 and TIMM10 are associated with significantly reduced overall survival. **B.** Note that that high transcript levels of NQO1 are associated with significantly reduced overall survival.

Firstly, we interrogated the utility of the behavior of mitochondrial chaperones and mitochondrial membrane proteins. Table 4 and Figure 3A both show that SLC25A5 and TIMM10 have significant prognostic value, with hazard-ratios of 2.67 and 2.63, respectively. Other members of the SLC25A, TIMM, TOMM and VDAC families also had prognostic value. Mitochondrial-related antioxidant proteins (NQO1 and SOD2), as well as mitochondrial creatine kinase, also had significant value (summarized in Table 4 and Figure 3B).

**Table 4:** Prognostic value of chaperones, mitochondrial membrane proteins, anti-oxidants and creatine kinase

Gene Probe ID	Symbol	Hazard-Ratio	Log-Rank Test
**Chaperones/HSPs**			
200691_s_at	HSPA9	1.77	0.047
**Membrane Proteins**			
200955_at	IMMT	2.61	0.002
218408_at	TIMM10	2.63	0.0008
201821_s_at	TIMM17A	2.46	0.003
217981_s_at	TIMM10B	1.94	0.05
218118_s_at	TIMM23	1.79	0.05
201519_at	TOMM70A	2.28	0.005
211662_s_at	VDAC2	2.32	0.01
208845_at	VDAC3	2.07	0.01
208846_s_at	VDAC3	1.96	0.048
200657_at	SLC25A5	2.67	0.0008
221020_s_at	SLC25A32	1.98	0.05
**Anti-Oxidant Proteins**			
201468_s_at	NQO1	3.48	0.001
210519_s_at	NQO1	2.37	0.006
215223_s_at	SOD2	1.82	0.048
**Mitochondrial Creatine Kinase**			
205295_at	CKMT2	2.27	0.0035

**Figure 4:**
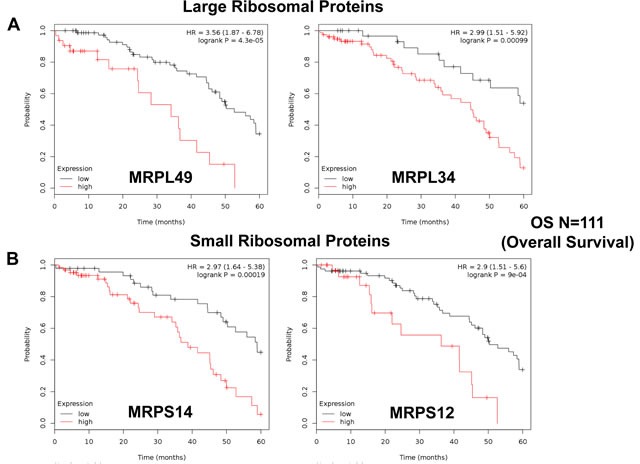
Mitochondrial ribosomal proteins (MRPs) are associated with poor clinical outcome in ovarian cancer patients **A.** Note that high transcript levels of MRPL49 and MRPL34 predict significantly reduced overall survival. **B.** Similarly, high transcript levels of MRPS14 and MRPS12 predict significantly reduced overall survival.

Next, we carefully examined the prognostic value of mitochondrial ribosomal proteins (MRPs). They functionally control the biosynthesis of essential components of the OXPHOS complexes, driving mitochondrial biogenesis (Table 5). Ten members of the large subunit (MRPLs) showed significant prognostic value, with hazard-ratios between 3.56 and 1.90. Interestingly, MRPL49 had the best prognostic value. Eleven different members of the small subunit (MRPSs) showed significant prognostic value, with hazard-ratios between 2.90 and 1.88. In summary, twenty-one different MRPs all predicted poor overall survival. Kaplan-Meier curves for representative examples are shown in Figure 4, panels A & B.

**Table 5:** Prognostic value of mitochondrial ribosomal proteins

Gene Probe ID	Symbol	Hazard-Ratio	Log-Rank Test
**Large Ribosomal Subunit**			
201717_at	MRPL49	3.56	4.3e-05
221692_s_at	MRPL34	2.99	0.001
218890_x_at	MRPL35	2.48	0.002
213897_s_at	MRPL23	2.48	0.01
217907_at	MRPL18	2.36	0.006
218281_at	MRPL48	2.29	0.007
222216_s_at	MRPL17	2.17	0.007
217980_s_at	MRPL16	2.17	0.008
219162_s_at	MRPL11	2.14	0.02
218105_s_at	MRPL4	1.90	0.03
**Small Ribosomal Subunit**			
203800_s_at	MRPS14	2.97	0.0002
204331_s_at	MRPS12	2.90	9e-04
210008_s_at	MRPS12	2.46	0.0035
221688_s_at	MRPS4	2.88	0.002
219819_s_at	MRPS28	2.64	0.0008
218001_at	MRPS2	2.15	0.01
219220_x_at	MRPS22	2.13	0.025
218654_s_at	MRPS33	2.05	0.02
217942_at	MRPS35	2.05	0.03
212604_at	MRPS31	2.02	0.02
221437_s_at	MRPS15	1.88	0.05

**Figure 5:**
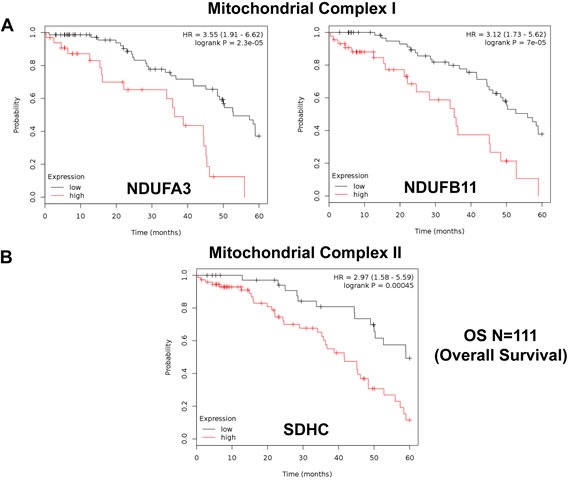
Mitochondrial complex I and II proteins are associated with poor clinical outcome in ovarian cancer patients **A.** Note that high levels of NDUFA3 and NDUFB11 predict significantly reduced overall survival. **B.** Similarly, high levels of SDHC predict significantly reduced overall survival.

**Figure 6:**
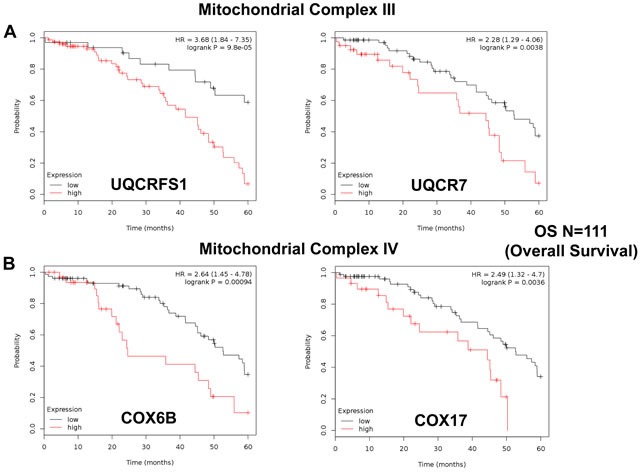
Mitochondrial complex III and IV proteins are associated with poor clinical outcome in ovarian cancer patients **A.** Note that high levels of UQCRFS1 and UQCR7 predict significantly reduced overall survival. **B.** Similarly, high levels of COX6B and COX17 predict significantly reduced overall survival.

**Figure 7:**
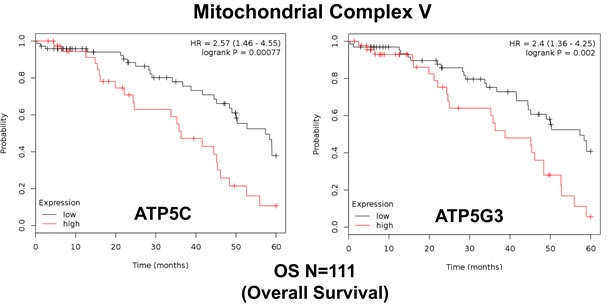
Mitochondrial complex V proteins are associated with poor clinical outcome in ovarian cancer patients Note that high levels of ATP5C and ATP5G3 predict significantly reduced overall survival.

Similarly, we also determined the prognostic value of key components of the OXPHOS complex. These results are summarized in Table 6. Surprisingly, 52 different gene probes for the OXPHOS complexes showed hazard-ratios between 3.68 and 1.76. Complex I had the most subunits with significant prognostic value (21 in total). However, UQCRFS1 (complex III) had the best individual prognostic value (HR = 3.68; *p* = 9.8e-05). NDUFA3 (complex I) also showed significant prognostic value (HR = 3.55; *p* = 2.3e-05). Kaplan-Meier curves for members of complex I and II are shown in Figure 5A & 5B, while results with members of complex III and IV are shown in Figure 6A & 6B. Results with complex V are shown in Figure 7.

**Table 6:** Prognostic value of mitochondrial OXPHOS complexes

Gene Probe ID	Symbol	Hazard-Ratio	Log-Rank Test
**Complex I**			
218563_at	NDUFA3	3.55	2.3e-05
218320_s_at	NDUFB11	3.12	7e-05
201740_at	NDUFS3	2.93	0.001
218200_s_at	NDUFB2	2.60	0.001
203371_s_at	NDUFB3	2.56	0.0008
203189_s_at	NDUFS8	2.43	0.002
218201_at	NDUFB2	2.43	0.002
203613_s_at	NDUFB6	2.43	0.008
202000_at	NDUFA6	2.43	0.0015
202785_at	NDUFA7	2.30	0.01
220864_s_at	NDUFA13	2.25	0.006
209303_at	NDUFS4	2.20	0.009
218160_at	NDUFA8	2.16	0.008
203190_at	NDUFS8	2.15	0.01
202941_at	NDUFV2	2.13	0.02
208714_at	NDUFV1	2.07	0.03
209224_s_at	NDUFA2	2.03	0.044
211752_s_at	NDUFS7	1.98	0.02
217860_at	NDUFA10	1.95	0.037
202298_at	NDUFA1	1.91	0.03
208969_at	NDUFA9	1.89	0.26
201966_at	NDUFS2	1.86	0.035
**Complex II**			
210131_x_at	SDHC	2.97	0.0005
202004_x_at	SDHC	2.78	0.0005
202675_at	SDHB	1.83	0.04
**Complex III**			
208909_at	UQCRFS1	3.68	9.8e-05
201568_at	UQCR7	2.28	0.004
209065_at	UQCR6	2.12	0.04
202090_s_at	UQCR	1.86	0.04
212600_s_at	UQCR2	1.76	0.047
**Complex IV**			
201441_at	COX6B	2.64	0.0009
203880_at	COX17	2.49	0.004
203858_s_at	COX10	2.47	0.002
211025_x_at	COX5B	2.34	0.004
202343_x_at	COX5B	2.32	0.004
202110_at	COX7B	2.30	0.02
218057_x_at	COX4NB	2.08	0.01
202698_x_at	COX4I1	1.89	0.03
201119_s_at	COX8A	1.87	0.04
204570_at	COX7A	1.76	0.05
**Complex V**			
208870_x_at	ATP5C	2.57	0.0008
213366_x_at	ATP5C	2.44	0.002
205711_x_at	ATP5C	2.08	0.01
207507_s_at	ATP5G3	2.40	0.002
210453_x_at	ATP5L	2.35	0.003
208746_x_at	ATP5L	2.24	0.005
207573_x_at	ATP5L	2.20	0.006
208972_s_at	ATP5G	2.15	0.007
207508_at	ATP5G3	2.12	0.01
202961_s_at	ATP5J2	1.91	0.02
217848_s_at	PPA1	1.89	0.03
202325_s_at	ATP5J	1.78	0.05

### Three new mitochondrial gene signatures for predicting overall survival, recurrence and the response to therapy

To significantly amplify the prognostic power of these unique mitochondrial markers, we then combined the most promising markers and to derive three new mitochondrial gene signatures.

**Figure 8:**
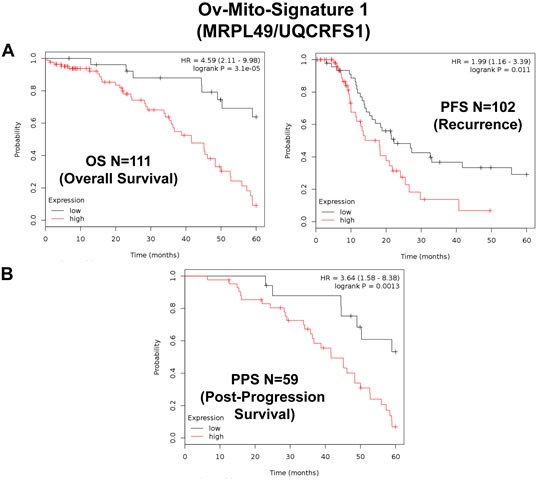
Ov-Mito-Signature 1 predicts patient outcome in ovarian cancer patients Note that high levels of Ov-Mito-Signature 1 (MRPL49/UQCRFS1) effectively predicts overall survival (OS), progression-free survival (PFS) and post-progression survival (PPS). OS and PFS are shown in panel A. PPS is shown in panel B.

**Figure 9:**
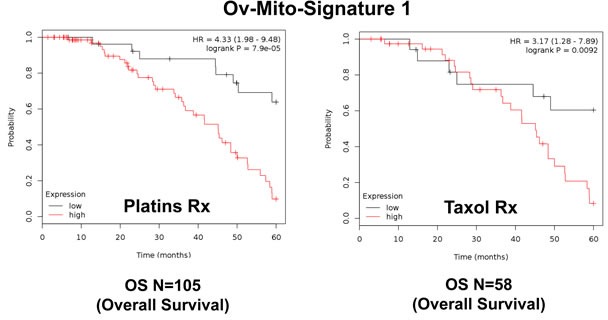
Ov-Mito-Signature 1 predicts the response to therapy in ovarian cancer patients Note that high levels of Ov-Mito-Signature 1 (MRPL49/UQCRFS1) effectively predicts drug-resistance and treatment failure, illustrated here as overall survival. Results with Platin and Taxol therapy (Rx) are shown.

**Figure 10:**
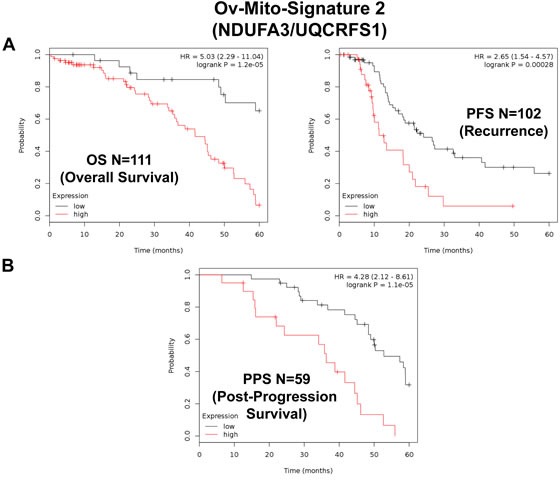
Ov-Mito-Signature 2 predicts patient outcome in ovarian cancer patients Note that high levels of Ov-Mito-Signature 2 (NDUFA3/UQCRFS1) effectively predicts overall survival (OS), progression-free survival (PFS) and post-progression survival (PPS). OS and PFS are shown in panel A. PPS is shown in panel B.

**Figure 11:**
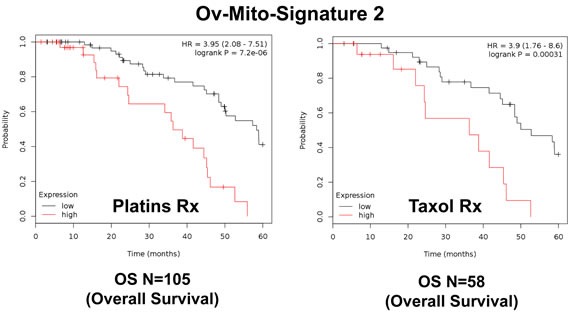
Ov-Mito-Signature 2 predicts the response to therapy in ovarian cancer patients Note that high levels of Ov-Mito-Signature 2 (NDUFA3/UQCRFS1) effectively predicts drug-resistance and treatment failure, illustrated here as overall survival. Results with Platin and Taxol therapy (Rx) are shown.

**Figure 12:**
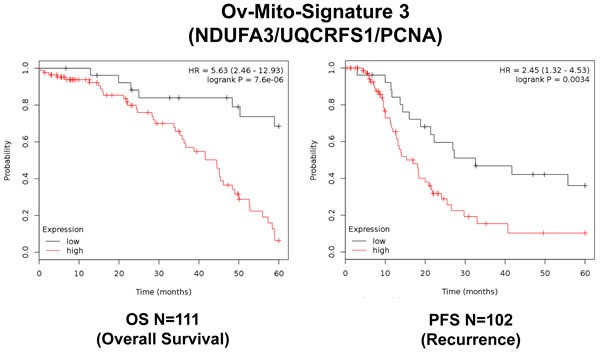
Ov-Mito-Signature 3 predicts patient outcome in ovarian cancer patients Note that high levels of Ov-Mito-Signature 3 (NDUFA3/UQCRFS1/PCNA) effectively predicts overall survival (OS), and progression-free survival (PFS).

**Figure 13:**
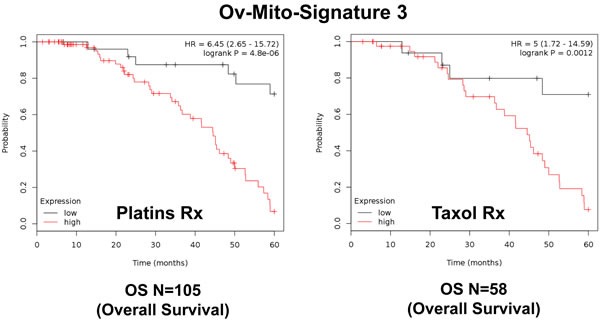
Ov-Mito-Signature 3 predicts the response to therapy in ovarian cancer patients Note that high levels of Ov-Mito-Signature 3 (NDUFA3/UQCRFS1/PCNA) effectively predicts drug-resistance and treatment failure, illustrated here as overall survival. Results with Platin and Taxol therapy (Rx) are shown.

**Figure 14:**
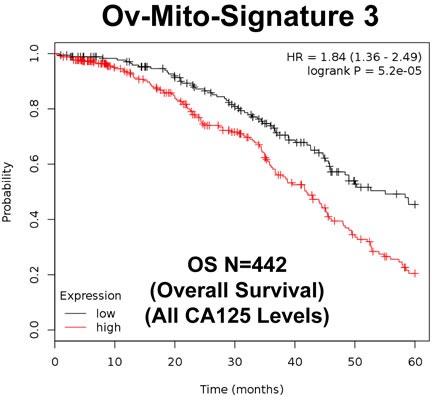
Ov-Mito-Signature 3 predicts patient outcome in ovarian cancer patients Note that high levels of Ov-Mito-Signature 3 (NDUFA3/UQCRFS1/PCNA) effectively predicts overall survival (OS), in a larger group of ovarian cancer patients (*N* = 442).

Ov-Mito-Signature-1 contains 2 genes (MRPL49/UQCRFS1). One component is an MRPL, while the other is part of the OXPHOS machinery (complex III). Ov-Mito-Signature-2 also consists of 2 genes (NDUFA3/UQCRFS1). Both components are part of the OXPHOS machinery (complexes I and III). In addition, Ov-Mito-Signature-3 consists of 3 genes (NDUFA3/UQCRFS1/PCNA), namely 2 mitochondrial genes and a proliferative marker (PCNA) (See Tables 7-9). K-M curves for these three signatures are shown in Figures 8-14.

**Table 7:** Prognostic value of ovarian mitochondrial signature 1

Gene Probe ID	Symbol	Hazard-Ratio	Log-Rank Test
208909_at	UQCRFS1	3.68	9.8e-05
201717_at	MRPL49	3.56	4.3e-05
**Combination**		**4.59**	**3.1e-05**

**Table 8:** Prognostic value of ovarian mitochondrial signature 2

Gene Probe ID	Symbol	Hazard-Ratio	Log-Rank Test
208909_at	UQCRFS1	3.68	9.8e-05
218563_at	NDUFA3	3.55	2.3e-05
**Combination**		**5.03**	**1.2e-05**

**Table 9:** Prognostic value of ovarian mitochondrial signature 3

Gene Probe ID	Symbol	Hazard-Ratio	Log-Rank Test
208909_at	UQCRFS1	3.68	9.8e-05
218563_at	NDUFA3	3.55	2.3e-05
201202_at	PCNA	2.85	0.0003
**Combination**		**5.63**	**7.6e-06**

Importantly, Ov-Mito-Signature-1 yielded a significantly improved hazard-ratio for overall survival of 4.59 (*p* = 3.1e-05) (Table 7 and Figure 8A, left). It was also highly predictive for progression-free survival (Figure 8A, right) and post-progression survival (Figure 8B), in the same group of patients. In addition, it effectively predicted the response to chemotherapy and treatment failure, in patients that received “Platin-derivatives” or “Taxol” (Figure 9).

Similarly, Ov-Mito-Signature-2 showed a hazard-ratio for overall survival of 5.03 (*p* = 1.2e-05) (Table 8 and Figure 10A, left). Ov-Mito-Signature-2 was also highly predictive for progression-free survival (Figure 10A, right) and post-progression survival (Figure 10B). Also, it effectively predicted the response to chemotherapy (Figure 11).

As such, both of these Ov-Mito-Signature(s) were a dramatic improvement over individual mitochondrial biomarkers, as well as CA125 and PCNA (Tables 1 & 3; Figure 2).

To further improve the predictive value of Ov-Mito-Signature-2, we next added the proliferative marker PCNA, to create Ov-Mito-Signature-3. The robust nature of Ov-Mito-Signature-3 is highlighted in Table 9 and Figures 12-14, which shows a hazard-ratio of 5.63 (*p* = 7.6e-06). Ov-Mito-Signature-3 was also the most effective in predicting the response to therapy (Figure 13). Importantly, Ov-Mito-Signature-3 retained its prognostic value in a larger group of serous ovarian cancer patients (*N* = 442), without restricting our analysis to patients with low serum CA125 levels (Figure 14).

## DISCUSSION

### Understanding CSCs, telomerase and mitochondrial activity: targeting ovarian cancer with doxycycline and/or palbociclib

The exact functional role of telomerase activity in ovarian cancer stem cell (CSC) propagation remains largely unknown. Recently, to address this issue, we indirectly monitored telomerase activity, by linking the hTERT-promoter to eGFP [6, 7]. Using SKOV3 ovarian cancer cells, stably-transfected with the hTERT-GFP reporter, we then used GFP-fluorescence to fractionate these cell lines into GFP-high and GFP-low populations. We functionally compared the phenotype of these GFP-high and GFP-low cell sub-populations. Importantly, we showed that ovarian cancer cells with higher telomerase activity (GFP-high) are energetically-activated, with increased mitochondrial OXPHOS and glycolysis [6]. This was confirmed by unbiased label-free proteomics analysis. A sub-population of SKOV3 cells with high telomerase activity showed i) increased “stemness” (3D-spheroid formation) and ii) enhanced cell migration (Boyden-chamber assay). These cellular phenotypes were halted by inhibitors of energy-metabolism, targeting either OXPHOS or glycolysis, or by using doxycycline, a clinically-approved antibiotic, that inhibits mitochondrial biogenesis [6, 7].

Telomerase activity also determined the ability of hTERT-high ovarian CSCs to proliferate, as determined by monitoring DNA-synthesis. Use of Palbociclib, a CDK4/6 inhibitor (an FDA-approved drug) specifically blocked ovarian CSC propagation, with an IC-50 of ∼100 nM [6]. Thus, telomerase-high ovarian CSCs are the most energetically-activated, migratory and proliferative cell sub-population [6]. These findings suggest a mechanistic interpretation for why long telomere length (a specific marker of high telomerase activity) is strictly correlated with metastasis disease progression and poor outcome in ovarian tumors and other cancer types [8, 9].

As such, elevated telomerase activity may “fuel” the propagation of ovarian CSCs

by activating mitochondrial biogenesis, ultimately leading to poor clinical outcome. These observations may help explain why combining mitochondrial markers, together with the proliferation marker PCNA, so significantly increased the prognostic value of this Ov-Mito-Signature.

### Employing mitochondrial markers and mito-signatures, as companion diagnostics for treatment stratification: implications for drug re-purposing

In support of our current hypothesis, integrating telomerase activity with increased mitochondrial function, we demonstrate that a sub-set of mitochondrial gene transcripts are able to predict survival in serous ovarian cancer patients, with optimal de-bulking. As such, these particular mitochondrial markers could ultimately be used to select high-risk ovarian cancer patients at diagnosis, up to 5 years in advance, for close monitoring. As such, our results provide an excellent justification for the therapeutic targeting of mitochondria in ovarian cancer cells, to improve patient survival.

**Figure 15:**
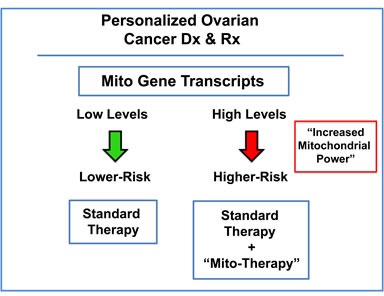
Ovarian cancer: mitochondrial-based companion diagnostics for personalized cancer therapy In this diagram, mitochondrial-based diagnostics would be used to separate ovarian cancer patients into higher-risk and lower-risk groups. Then, patients with high levels of mitochondrial markers in their primary tumor (“bad prognosis”) would be treated with mitochondrial-based therapies (such as “Doxycycline”), as an add-on to the standard of care, to prevent tumor progression and increase overall survival.

In this new paradigm, high-risk patients would be identified at diagnosis by the over-expression of mitochondrial mRNA transcripts in their ovarian tumors (Figure 15). As a consequence, these patients could then be treated with certain FDA-approved drugs (e.g., Doxycycline or Palbociclib; together with the standard of care), to improve overall survival. These therapeutics have been previously documented to halt the proliferation of the ovarian CSC population [6].

FDA-approved antibiotics can safely prevent mitochondrial biogenesis and/or OXPHOS as a manageable, off-target, “side-effect” [6, 7, 10–16]. These antibiotics include the tetracyclines, the erythromycins, as well as pyrvinium pamoate, atovaquone, and bedaquiline [10, 11, 13, 14]. For example, the new mitochondrial markers and Mito-Signatures we have discovered, could be used as companion diagnostics, for re-purposing these FDA-approved drugs as novel anti-cancer agents. More specifically, this would facilitate the ability of medical oncologists to identify the correct patient sub-population for new phase II clinical trials for drug re-purposing/re-positioning in serous ovarian patients, as an add on to conventional chemo-therapy (e.g., platins and taxol).

### Mitochondrial markers and mito-signatures: implications for new drug discovery

The three new Mito-Signatures that we developed may also be useful for selecting new “druggable” targets for new drug development, to prevent treatment failure and improve overall survival. As a consequence of our K-M analyses, the mitochondrial ribosome would be an attractive new target for developing novel inhibitors of mitochondrial protein translation in cancer cells; similarly, mitochondrial chaperones, the OXPHOS complexes and the mitochondrial ATP-synthase may also be suitable drug targets. Multiple members of these multi-subunit protein complexes show significant prognostic value, suggesting that modulation of their intrinsic activity may provide therapeutic benefits. Targeting of these large complexes would be predicted to suppress tumor recurrence and prevent disease progression in these serous ovarian cancer patients.

In addition, such mitochondrial markers could also be employed as companion diagnostics for novel therapies targeting either mitochondria or telomerase (hTERT) and/or cell proliferation, to select the high-risk sub-population of ovarian cancer patients, resulting in the necessary treatment stratification. In direct support of this assertion, we showed here that three different Mito-Signature(s) could be used to successfully identify the sub-population of high-risk ovarian cancer patients that failed “platin” or “taxol” based therapies. These results indicate that mitochondrial markers could be used to monitor and/or predict the response to therapy, specifically identifying patients at high-risk for treatment failure at diagnosis, up to 5 years in advance, even before therapy is initiated.

## METHOD OF ANALYSIS

Kaplan-Meier (K-M) Analyses. To perform K-M analysis on nuclear mitochondrial gene transcripts, we used an open-access online survival analysis tool to interrogate publically available microarray data from up to 1,435 ovarian cancer patients [5]. This allowed us to determine their overall prognostic value. For this purpose, we primarily analyzed 5-year follow-up data from serous ovarian cancer patients (stage III) that had optimal de-bulking (*N* = 111). Biased array data were excluded from the analysis. This allowed us to identify >100 nuclear mitochondrial gene probes, with significant prognostic value. Hazard-ratios for overall survival (OS), progression free survival (PFS; recurrence) and post-progression survival (PPS) were calculated, at the best auto-selected cut-off, and p-values were calculated using the logrank test and plotted in R [5]. K-M curves were also generated online using the K-M-plotter (as high-resolution TIFF files), using univariate analysis:

http://kmplot.com/analysis/index.php?p=service&cancer=ovar.

This allowed us to directly perform *in silico* validation of these mitochondrial biomarker candidates. The 2012 version of the database was originally utilized for all these analyses; however, virtually identical results were also obtained with the 2015 and 2017 versions.

## Abbreviations

CSCs: cancer stem-like cells
HR: hazard ratio
K-M: Kaplan-Meier
MRPL: mitochondrial ribosomal proteins, large subunit
MRPS: mitochondrial ribosomal proteins, small subunit
N: number of patients in a given data set
OS: overall survival
OXPHOS: oxidative phosphorylation (mitochondrial respiration)
PPS: post progression survival
PFS: progression-free survival
